# The Institutional Library

**Published:** 1915-06-05

**Authors:** 


### THE INSTITUTIONAL LIBRARY.

### From the Russian Trenches.

FIELD HOSPITAL AND FLYING COLUMN. By VIOLETTA THURSTAN. (Putnams. 8vo. Pp. 183. 2s. 6d. net.)

THIS brightly written volume is a record of the experiences of an English Red Cross sister in Belgium during the early days of the war, and afterwards in Russia, whither she managed to find her way when the Germans made life unbearable for all English doctors and nurses in Flanders. It is written very simply. There are no heroics in the book, no purple patches. It is a pleasant relief, therefore, after the surfeit of exaggerated sentimentality we have been suffering from, poured forth by amateurs up against the grim realities of life for the first time. For this is the work of a professional; and to those who know and have worked in similar surroundings it rings true.

Yet for all its quietness the book unfolds a very heroic tale—a tale of work, never-ending work, under dangers and difficulties, under disappointments, under brutal German obstruction, that loses nothing—that gains indeed—from the fact that the author does not seem to realise herself how very wonderful it was. We follow her in her account of her work in Brussels and Charleroi with ever-increasing interest. She makes us feel with her the bitterness of her imprisonment in Germany, the joy of her deliverance into hospitable Denmark, and the delight of getting into harness again amid sympathetic surroundings with our great ally Russia. The descriptions of her experiences in Warsaw, in Lodz, and in the Russian trenches are dramatic in the extreme. The account given of the work of the famous flying column of ambulance motors is particularly well done. Quotations from the book would give no adequate idea of its merits. To be appreciated it must be read as a whole. We can recommend it unreservedly.

### Chronicles of an Old Campaigner.

A CAMPAIGN AGAINST CONSUMPTION. A Collection of Papers Relating to Tuberculosis. By ARTHUR RANSOME, M.D., F.R.C.P., F.R.S. (Cambridge: At the University Press, 1915. Price 10s. 6d.)

DURING the past thirty years the public has been regularly talked at on the subject of the prevention and extermination of consumption. At one time the plan suggested for the abolition of phthisis was the apparently simple one of ostracising all the victims of the disease and the burning of their belongings; later on it was thought that an excellent plan would be to cure all the early cases in beautiful sanatoria. But the advanced cases were to be left unfettered in their efforts to infect the coming generation, and were thus to keep up the supply of "early" cases, whose treatment was so successful that shortly the words "sanatorium treatment" became almost synonymous with "cure guaranteed." With the advent of the National Insurance Act and its wildly boomed sanatorium benefit the early cases were overwhelmed in the welter of insured consumptives, who, by insistent clamour for their "rights," managed to get sent to the makeshift "hotels" promised by the politicians. In point of fact, this was simply the carrying out of the principle of the segregation of the advanced case, which should, of course, be one of the main objectives in any campaign against tuberculosis, though the segregation need not necessarily be institutional or in colonies.

Of public-health problems of the present day, excluding for the moment everything pertaining to the state of war, the control of tuberculosis still holds the most prominent position, having displaced, at any rate for the time, the claims to popularity of medical inspection of school children. Tuberculosis officers are now having their innings, and in the great game of finding the early cases many sorts and conditions of men, women, and children have been enabled to obtain highly beneficial holidays, and medical officers have been able to produce most satisfactory statistics. The diagnosis, too, of the early stages of pulmonary tuberculosis has no doubt been greatly "advanced," and the experts will possibly soon be able to ascertain the presence of tubercle in the lung in its fœtal if not in its embryonic stage. The number of methods of treatment which have come into and gone out of fashion is legion, but, nevertheless, the death-rate of phthisis has, fortunately, fallen by half during the last forty years. Apparently this result has been obtained more or less independently of direct methods of attack, and may be ascribed in part to general sanitary reforms and to a gradual rise in the resistance of the population.

To realise what our predecessors have done is surely a profitable experience, and a study of the current opinions and methods of our immediate forerunners may afford some salutary lessons to those who are only entering upon a new phase of an old campaign. To whom could we turn for a better opportunity to indulge in this exercise than to Dr. Arthur Ransome, whose entrance into the campaign against consumption dates back to the year 1860? He has been a fairly "constant contributor" of papers on the subject of tuberculosis, and has been well advised to republish in book form some of his writings, which deal with the subject both from the public health and scientific points of view. These papers, now published by the University Press, Cambridge, cover a period extending from 1881 to 1913, and are divided into four sections. The first section includes papers of a practical character dealing with consumption in a general way. The first paper is particularly interesting, for it represents a health lecture delivered in Manchester before the discovery of the bacillus of tuberculosis. The lecture even to-day would be justly described as very "sound"; the causes considered include, naturally, "the obvious one of inheritance," but the chief cause is given as "foul air"—a statement as true to-day as it was when the lecture was given. Medical practice, even in the 'eighties, did not encourage, it must be feared, the admission of fresh air to sick rooms as freely as might have been expected, seeing how many ancient and modern authorities could then have been cited as enjoining fresh air for the treatment of phthisis. An old doggerel rhyme of the eighteenth century quoted in the lecture is sufficiently interesting for repetition here :—

> "If consumption cured can be,
> Which is a mighty rarity;
> These three things you must prepare,
> Milk, traumatics, and fresh air."

Other papers in this section foreshadow a crusade against tuberculosis, and two quite modern addresses deal with the duties of the State in regard to this disease and the need for co-ordinating anti-tuberculosis measures. Articles well worth reprinting on the conditions of infection find a place in the second section, while another series of papers, relating to various researches, is a useful reminder of the ever-changing nature of the treatments so zealously advocated at one time or another.

The fourth and last section contains papers chiefly statistical, and includes one entitled "Tuberculosis and Leprosy: a Parallel and a Prophecy." This interesting essay calls attention to the remarkable likeness between leprosy and tuberculosis, and from a consideration of the circumstances attending the decline of the former disease the writer ventured to hope for a comparatively speedy extinction of phthisis. The steady fall of the curves representing the death-rates from phthisis of males and females in England gave at that time great support to that hope, but, as we know, statistics of death-rates are open to considerable errors and modifications, and at the present time the curve for male deaths is falling less rapidly than that for females, and both are less steep than previously.

Since 1860, when Dr. Ransome started a weekly register of all new cases of certain diseases, of which phthisis was one, to the present day, when tuberculosis has become one of the compulsorily notifiable diseases, so much has been said, written, and done that an opportunity of reviewing the past is not to be despised, and hence this book is worthy of the consideration of medical officers of health and tuberculosis officers. Perhaps before the end of this century some future medical historian will collect papers and lectures on tuberculin, or nascent iodine treatment, and possibly on the establishment of pneumothorax surgeries and nitrogen injection stations! It may be hoped that such a collection will be as interesting as these present chronicles of an old campaigner.

### "The Knowledge of an Anatomist and the Experience of a Physician."

Medical Applied Anatomy. By T. B. Johnston, M.B., Ch.B. (London: A. and C. Black, Ltd. 1915. Pp. 436+xiv. Price 7s. 6d. net.)

In writing this book Dr. Johnston has undertaken a very difficult task. The difficulty is due not to any defect in the supply of material, but rather to the embarrassment consequent on excess. What is anatomy? and what is medicine? sound very plain questions until you try to write a book to explain the mutual relations of the two, and then all sorts of awkward problems arise. It is necessary, for example, to try to settle what amount of anatomy can reasonably be called medical, for until this is defined your book may be hard to distinguish from any one of a score of competitors which profess only anatomy. And in making the double venture it is readily possible to miss both targets. Then, again, ought physiology to be excluded? The title implies such exclusion, but how can the writer, with the physician as his aim, relate the hard facts of structure and leave the functional working of the apparatus in severe silence? Still remains the task of dividing those artificial rivals known as medicine and surgery. Now we will not pretend to say that Dr. Johnston has satisfactorily settled all these issues, or that he has been perfectly happy in his efforts to this end. Broadly speaking, we should decide that his anatomy is stronger than his medicine. We will not suggest that there is too much of the former, but there is certainly too little of the latter. Take two or three examples. After a description of the structure of the eyeball we are told of the use of the ophthalmoscope, which, we learn, gives "important information . . . in cases of early arterio-sclerosis." Now, we could have understood the total omission of the story of the ophthalmoscope, but why a mere fragment should be related and all the rest ignored is hard to explain. Again, there is a discussion of polygraphic tracings, which if not anatomy is certainly medicine, but the only statement relative to the ventricular type of venous pulse is that it is due to tricuspid incompetence (!); the term "auricular fibrillation" receives no recognition. And, once more, in a comparison of the effects of lesions in the upper and lower motor neurones respectively there is not a single reference to the several conditions of the reflex apparatus or to the display of these conditions in the state of the tendon phenomena. Take these selections, and is it not fair to say that the virtues of the book must be sought in its anatomical rather than in its medical achievements? This is by no means to say that the book has not many excellencies. Indeed, quite the contrary is the case. It is both well written and well illustrated, and we can readily believe that the practitioner who wishes to revive his anatomical knowledge will find it distinctly helpful in the direction of his desires, while the occasional clinical applications will come to him as a pleasing experience. But we doubt whether he will agree that all cerebral tumours are progressive, or that in syringomyelia the "motor paralysis is always of the upper motor neurone type."

Perhaps it is impossible for any one man to write a satisfactory book on medical anatomy, whilst if two attempted the task they would be very like to quarrel. An anatomist cannot suffer the exclusion of much that is dear to his heart merely because the physician says it is of no practical value, while the physician rebels against details that to the anatomist seem the very joy of life. If, however, Dr. Johnston can find a physician of the necessary mildness of temper and willing to take part in the task, we think he could much improve his book when it comes to a second edition, and could make it more closely correspond with the title which it carries. We have the fullest appreciation of his effort and of the measure of success he has obtained; in so far as he has failed, failure was perhaps inherent in his task. The course here suggested we offer with great respect and with a full recognition of the truth that it is easier to criticise a book than to write it. Especially is this true in reference to a book needing both the knowledge of an anatomist and the experience of a physician. One minor point and we have done. Is "Argyll-Robertson" the correct name? The hyphen nowadays is commonly seen, but we do not recollect it in earlier days. As this book comes from Edinburgh, it is all the more necessary that accuracy should mark the presentation of one of her great names.

